# Case report: Neonatal case of intrauterine gastrointestinal bleeding with suspected cow's milk allergy or neonatal transient eosinophilic colitis

**DOI:** 10.3389/fped.2023.1213782

**Published:** 2023-06-27

**Authors:** Keisuke Hoshi, Takeo Mukai, Keiichi Kumasawa, Naoto Takahashi

**Affiliations:** ^1^ Department of Pediatrics, The University of Tokyo Hospital, Tokyo, Japan; ^2^ Department of Obstetrics and Gynecology, The University of Tokyo Hospital, Tokyo, Japan

**Keywords:** neonatal transient eosinophilic colitis, non-IgE-mediated gastrointestinal food allergy, food allergy, premature infants, fetal gastrointestinal bleeding, honeycomb sign

## Abstract

The patient was a female newborn. Ultrasonography performed at 35 weeks and 3 days of gestation revealed honeycomb-like dilatation and peri-intestinal strong echo patterns in the gastrointestinal tract. Nonreassuring fetal status was also diagnosed, leading to an emergency Cesarean section. The baby's birth weight was 2,127 g, whereas the Apgar 1 min and 5 min scores were 8 and 9, respectively. The amniotic fluid showed fecal and hematogenous turbidity. After delivery, there was hematogenous intragastric residue and defecation. Thereafter, the bloody intragastric residue and fecal discharge improved. Aggregations of eosinophils in the stool were observed, and gastrointestinal allergy was suspected. Enteral feeding with the hydrolyzed protein formula was initiated and symptoms did not recur. The allergen-specific lymphocyte stimulation test was positive for lactoferrin, and the patient was suspected with neonatal cow's milk allergy or neonatal transient eosinophilic colitis. After her condition stabilized, an oral challenge test was performed using breast milk without dairy products, and the test was negative. Gastrointestinal allergy is rare even *in utero*, and when gastrointestinal bleeding is suspected *in utero*, hemorrhagic or surgical gastrointestinal diseases should be ruled out first; however, the possibility of gastrointestinal allergy should also be kept in mind.

## 1. Introduction

Gastrointestinal allergy can be broadly classified into the following three categories based on the presence or absence of antigen-specific IgE: IgE-mediated, mixed, and non-IgE-mediated. Among them, those with gastrointestinal symptoms such as vomiting, diarrhea, melena, and poor weight gain in the neonatal period and infancy are considered non-IgE-mediated. Often, they are related to cellular immunity (1). Most of these diseases occur after suckling; however, we experienced a case of fetal distress due to intestinal honeycombing, dilatation, and intestinal retention *in utero*, in addition to hematemesis and bloody stools observed immediately after birth. Such neonatal and infantile gastrointestinal food allergy that occurs *in utero* is rare, and it is reported here along with the course of treatment.

## 2. Case presentation

**Figure 1:**
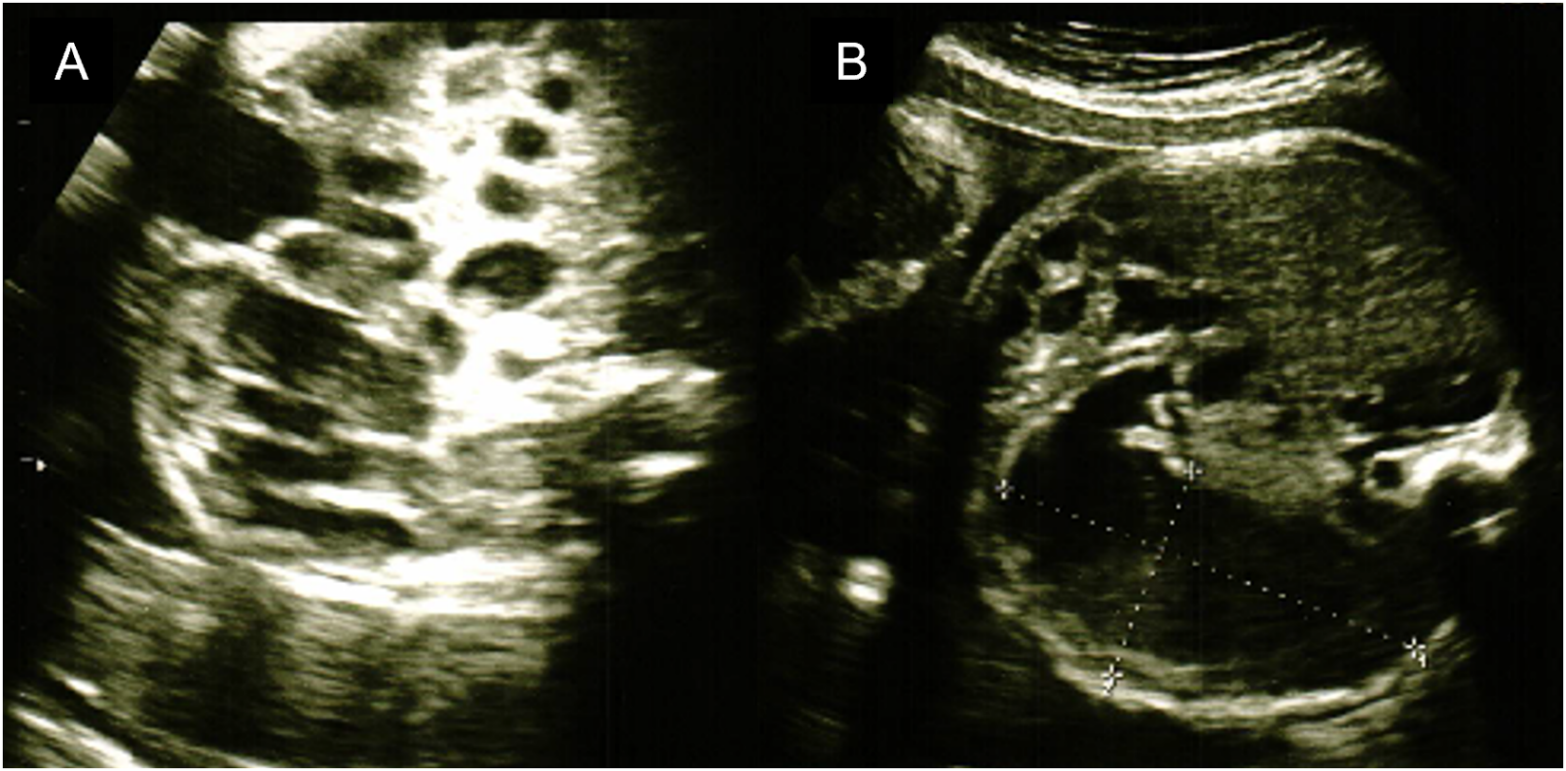
The fetal ultrasonography. (**A**) Image of honeycomb-shaped intestinal dilation. (**B**) Image of gastric bubble dilation.

**Figure 2:**
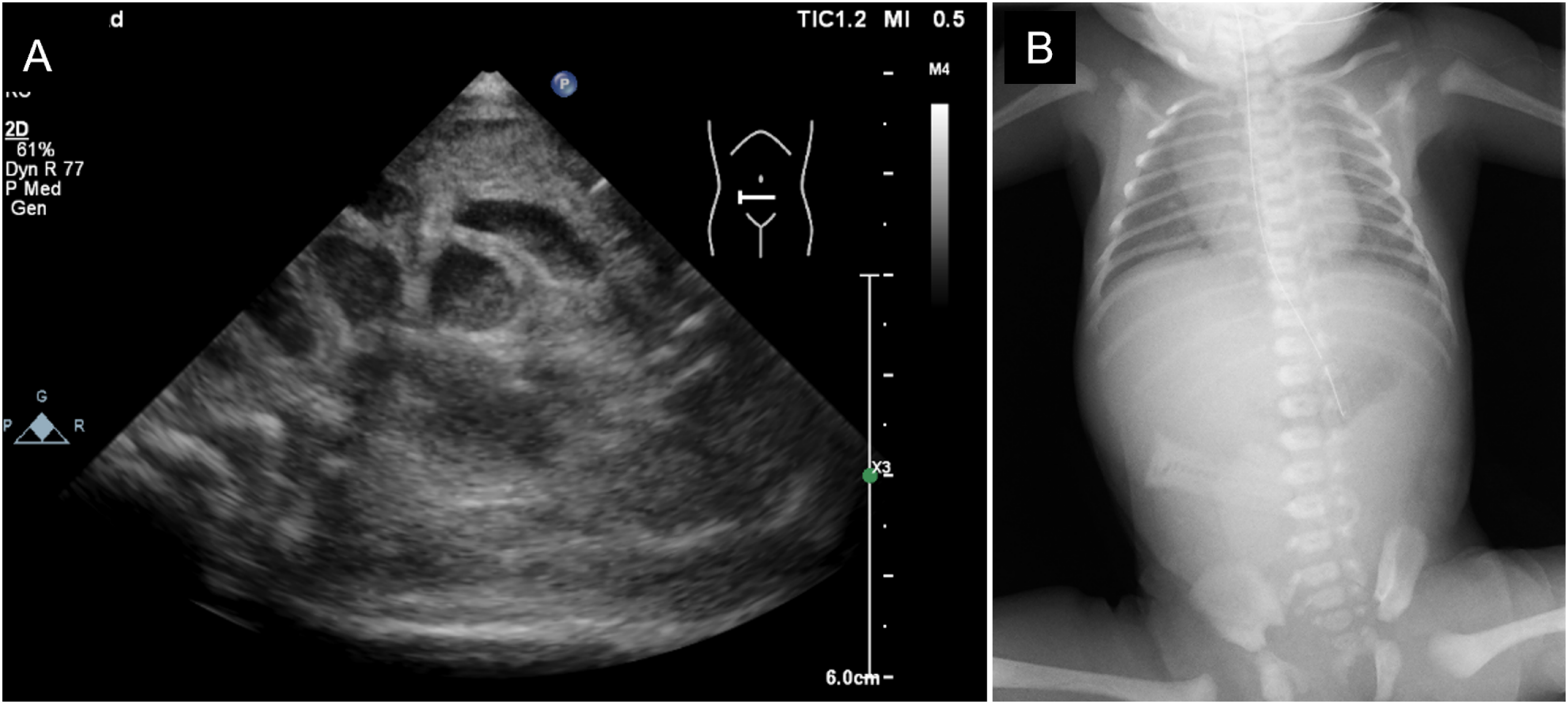
Imaging examination on admission. (**A**) Abdominal ultrasonography. (**B**) x-ray of the chest and abdomen.

The mother was a first-time mother, and the baby was conceived naturally. The mother enjoyed eating cheese both before and during the pregnancy. She noted decreased fetal movements at 31 weeks and 1 day, and increased abdominal tension and bloating at 31 weeks and 2 days. Ultrasonography revealed intestinal dilatation and peri-intestinal strong echo patterns in the fetus, and the mother was sent to our hospital on suspicion of fetal meconium peritonitis due to intestinal tract perforation. Ultrasonography performed on arrival at the hospital revealed fetal heartbeats and fetal movements, together with dilated gastric bubbles and honeycomb-shaped intestinal dilatations (Figures 1A,B). The amniotic fluid index (sum of four quadrant measurements of the deepest pocket in the vertical dimension with a normal range of 5–25 cm) was high (27 cm). The echo intensity of the gastric bubbles and amniotic fluid was biphasic, with areas of high intensity. The maximum systolic blood flow velocity of the middle cerebral artery was 71 cm/s, which suggested severe anemia. The cardiotocogram revealed the disappearance of baseline fetal heart rate changes. Based on the above findings, we suspected hydramnios and hematogenous amniotic fluid and determined that the nonreassuring fetal status was due to intestinal obstruction and impaired placental function. Therefore, an emergency Cesarean section was performed due to the nonreassuring fetal status. The amniotic fluid was brown and had a mixture of turbidity due to fecal material and old blood. The baby cried immediately after delivery and had no bradycardia. Immediately after birth, old bloody fluid was withdrawn from the oral cavity and stomach, and one minute after birth, watery old bloody stools were seen at the anus. The Apgar scores at 1 min and 5 min were 8 points and 9 points, respectively. The birth weight was 2,127 g, height was 45 cm, and the newborn's vitals were stable. There was no external malformations. Ultrasonography revealed dilatation of the small intestine, and chest radiography revealed little intestinal gas (Figures 2A,B). Peripheral blood examination revealed a low Hb level of 12.7 g/dl. The white blood cell count was 47,100/μl (Seg: 50.0%, Band: 12.0%, Eosino: 6.0%). Leukocytosis and eosinophilia were initially observed; however, the eosinophil count started to decrease thereafter. The patient's clotting profile was normal, PIVKA-II was 69 mAU/ml, and vitamin K deficiency was ruled out. Blood culture was negative; sputum and stool culture did not detect any significant bacteria either. The stool mucus test revealed eosinophil accumulation. The IgE level was 2 IU/ml (not elevated). The specific IgE antibody titers by CAP-Radioallergosorbent test method test was negative for milk antigens (α-lactoglobulin, β-lactoglobulin, milk, and casein, at <0.10 UA/ml each). Allergy testing by the allergen-specific lymphocyte stimulation test (ALST) was negative for kappa-casein (SI 0.79), positive for lactoferrin (SI 3.21), and negative for alpha-lactalbumin (SI 1.48). Bloody stools and intragastric residues decreased and improved with time after admission. Oral food challenge test of the extensively hydrolyzed protein formula (new MA-1, Morinaga Milk Industry Company, Ltd., Tokyo, Japan) was started at 3 days of age. After that, she underwent an oral food challenge test using breast milk without maternal intake of dairy product and was allowed to feed independently on breast milk without dairy products after 48 days of age. Her condition stabilized and she was discharged from the hospital at 67 days of age. She is currently corrected 11 months old, and no neurodevelopmental disorders are observed. This study was approved by the Institutional Review Board of the Ethics Committee of the University of Tokyo Hospital (approval ID: 2701).

## 3. Discussion

The prevalence of non-IgE-mediated gastrointestinal food allergy is not widely reported among gastrointestinal allergies; however, its incidence ranges from 0.13% to 0.72% (1). The lesions are widely distributed from the esophagus to the large intestine, with endoscopic presentations of eosinophilic esophagitis and gastroenteritis, and some histopathologic findings (including marked eosinophilic infiltration). In particular, in pathological subtypes presenting with eosinophilic infiltration of the upper gastrointestinal tract, there is increased expression of IL-5, IL-13, and CCL26 (2, 3). In the mucosa of the proctocolitis type (FPIAP), which is primarily characterized by melena, there is increased expression of CCL11 (eotaxin-1), CXCL-13, etc. (4). Therefore, the diagnosis must first be based on a thorough differentiation of the gastrointestinal symptoms. In the present case, the patient presented with melena and positive stool eosinophils, which suggested FPIAP; however, the patient also presented with bloody gastric residues, which may have been a complication of FPIES, with another possibility of subsequent acute gastric mucosal lesion. Other possibilities include neonatal transient eosinophilic colitis (NTEC), which is reported to present single bubble sign of stomach (5) alike the presenting case, but might lack of allergic mechanism, and acute gastric mucosal lesion, which might follow other causes. In any case, gastrointestinal hemorrhage of fetal onset without oral feeding is very rare. E. Ferretti et al. reported cases of premature milk allergy in preterm and low-birth-weight infants, and in both cases, the diagnosis was made after postnatal feeding (6). In the present case, no gastrointestinal endoscopy or biopsy was performed because of the infant's low-birth-weight and prematurity. In this case, the oral food challenge test by formula milk for diagnosis of non-IgE-mediated gastrointestinal food allergy should be considered for the infant.

Although IgE-mediated-allergic sensitization *in utero* has been discussed using IgE in umbilical cord blood (7, 8), the pathogenesis of the *in utero* sensitization of non-IgE-mediated gastrointestinal food allergy has not been reported in the past. This child developed the disease before suckling, and sensitization from breast milk was not involved. However, there are reports that lactoferrin is contained in amniotic fluid, and its concentration increases after infection or at 32 weeks of gestation. Therefore, a possible route of sensitization in the fetal period is enteral sensitization by drinking amniotic fluid containing this antigen (8, 9). No determinative test was performed before delivery; the intrauterine events were suspected of being caused by milk protein allergy. The limitation in discussing the present case is that no gastrointestinal endoscopy or biopsy was performed because of the low-birth-weight and prematurity of the infant, making it difficult to definitively determine with utmost certainty the type of disease the patient had.

It has also been reported that honeycomb changes and intestinal tract dilatation during the fetal period are observed in congenital chloride diarrhea (CCD) (10). CCD is a rare disease that causes frequent watery diarrhea due to impairments in the active transport of chloride (Cl^−^) in the terminal ileum and colon. In CCD, the fetal stage is characterized by maternal hydramnios, fetal intestinal dilatation, and watery diarrhea beginning immediately after birth, and it is associated hypochloremic metabolic alkalosis and elevated Cl^−^ levels in the stool. The postnatal course of CCD differs from that in the present case because it is a life-long disease involving diarrhea.

The honeycomb changes and dilatations of the intestinal tract in the fetus are generally thought to be the most commonly associated with gastrointestinal obstruction, Hirschsprung's disease, or other surgical diseases that require postnatal surgical treatment. However, it must be kept in mind that gastrointestinal food allergy, such as that in the present case, may also present with honeycomb-like changes and dilatations of the intestinal tract during the fetal period, resulting in nonreassuring fetal statuses.

## 4. Conclusion

We experienced a case of gastrointestinal food allergy or NTEC in which the patient presented with honeycomb changes, gastrointestinal tract dilatation, a nonreassuring fetal status during the fetal period, bloody stools, and intragastric residues after delivery. If gastrointestinal hemorrhage is suspected during the fetal period, surgical diseases such as neonatal melena, intestinal malrotation, and infectious diseases should be ruled out as part of the differential diagnosis. In such a case, it is necessary to eliminate the antigen and conduct a thorough examination.

## Data Availability

The original contributions presented in the study are included in the article, further inquiries can be directed to the corresponding author.
